# Real-World Outcomes of Limited Resection for Tumours Greater Than 20 mm in Non-Small Cell Lung Cancer

**DOI:** 10.1093/ejcts/ezaf322

**Published:** 2025-09-29

**Authors:** Shigeki Suzuki, Keisuke Asakura, Kyohei Masai, Kaoru Kaseda, Tomoyuki Hishida, Akio Kazama, Takao Shigenobu, Ryutaro Hanawa, Katsura Emoto, Yasunori Sato

**Affiliations:** Department of General Thoracic Surgery, Keio University School of Medicine, Tokyo 160-8582, Japan; Department of General Thoracic Surgery, Sagamihara Kyodo Hospital, Kanagawa 252-5188, Japan; Department of General Thoracic Surgery, Keio University School of Medicine, Tokyo 160-8582, Japan; Department of General Thoracic Surgery, Keio University School of Medicine, Tokyo 160-8582, Japan; Department of General Thoracic Surgery, Keio University School of Medicine, Tokyo 160-8582, Japan; Department of General Thoracic Surgery, Keio University School of Medicine, Tokyo 160-8582, Japan; Department of Pathology, Sagamihara Kyodo Hospital, Kanagawa 252-5188, Japan; Department of General Thoracic Surgery, Saiseikai Utsunomiya Hospital, Tochigi 321-0974, Japan; Department of General Thoracic Surgery, Saiseikai Utsunomiya Hospital, Tochigi 321-0974, Japan; Department of Pathology, Keio University School of Medicine, Tokyo 160-8582, Japan; Department of Biostatistics, Keio University School of Medicine, Tokyo 160-8582, Japan

**Keywords:** non-small cell lung cancer, passive limited resection, local recurrence, surgical margin, consolidation-to-tumour ratio

## Abstract

**OBJECTIVES:**

Sublobar resection is an established surgical option for early-stage non-small cell lung cancer. However, evidence remains limited regarding its use for tumours >20 mm in real-world settings. We evaluated characteristics and outcomes of limited resection in this context and identified predictors of local recurrence.

**METHODS:**

We retrospectively analysed 165 patients with clinical stage I non-small cell lung cancer with tumours >20 mm who underwent limited resection between 2007 and 2017. Clinical, pathological, and radiological data were reviewed. The primary end-point was local recurrence, assessed using competing risk analysis. Overall survival and disease-free survival were estimated using Kaplan-Meier and Cox models.

**RESULTS:**

We analysed 165 patients with 13 local recurrence events. Among them, 146 (88.5%) had identifiable reasons for not undergoing lobectomy. Segmentectomy and wedge resection were performed in 59% and 41% of cases, respectively. Lymph node dissection was performed in all segmentectomies and in 20% of wedge resections. The 5-year overall and disease-free survival rates were 64.0% and 62.1%. Local recurrence occurred in 8%, more frequently after wedge resection than segmentectomy (13% vs 4%, *P* = .04). Solid-predominant tumours with a consolidation-to-tumour ratio greater than 0.5 accounted for 76% and were independently associated with lower disease-free survival (hazard ratio, 2.65; *P* = .05) and higher local recurrence (hazard ratio: infinite; *P* < .001). No local recurrence was observed in tumours with a ground-glass opacity-predominant pattern.

**CONCLUSIONS:**

Limited resection showed acceptable outcomes in lung cancers >20 mm, especially with ground-glass opacity; solid-predominant CT patterns were strongly linked to recurrence.

## Graphical abstract

**Figure:**
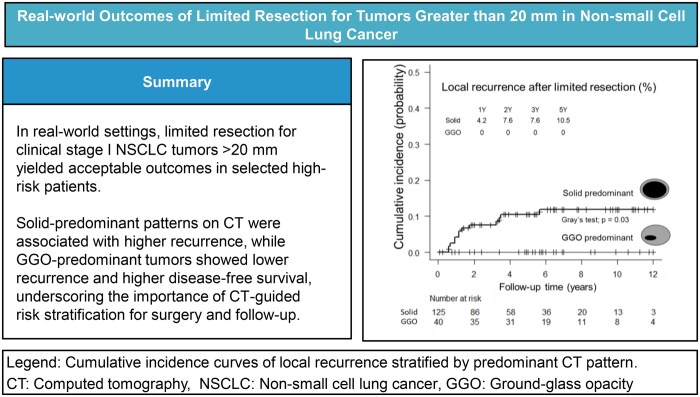

## INTRODUCTION

Sublobar resection is a viable surgical option for stage IA1 and IA2 non-small-cell lung cancer (NSCLC), and several studies have demonstrated that it offers overall survival (OS) outcomes comparable to those of lobectomy.^1–3^ However, lobectomy has remained the standard of care due to its lower incidence of locoregional recurrence.^4^

Passive limited resection, including segmentectomy and wedge resection, is typically performed in patients for whom lobectomy poses substantial medical risks. Because there is no official definition of high-risk, clinical decision-making is often based on factors such as advanced age, impaired pulmonary function, and comorbidities that may contraindicate standard surgery. To minimize surgical morbidity, lymph node dissection is often omitted in passive limited resections.

Wedge resection is associated with a lower risk of perioperative complications than segmentectomy but tends to yield shorter surgical margins.^5^^,^^6^ Therefore, segmentectomy is generally preferred when wedge resection is deemed inadequate to achieve appropriate margins. A multicentre randomized controlled trial conducted by the American College of Surgeons Oncology Group (ACOSOG) Z4032 evaluated whether adding intraoperative brachytherapy to sublobar resection could reduce local recurrence in high-risk operable patients with clinical stage I NSCLC (tumour ≤30 mm).^5^ The trial defined patients as high-risk if they met at least 1 major or 2 minor criteria. Major criteria were as follows: (1) forced expiratory volume in 1 second [FEV1] ≤50% predicted and (2) diffusing capacity of the lung for carbon monoxide ≤50% predicted. Minor criteria consisted of (1) age ≥75 years, (2) FEV1 51%-60%, (3) diffusing capacity of the lung for carbon monoxide 51%-60%, (4) pulmonary hypertension, (5) poor left ventricular function, (6) resting hypoxemia (partial pressure of oxygen ≤55 mmHg or peripheral oxygen saturation ≤ 88%), (7) partial pressure of carbon dioxide ≥45 mmHg, and (8) Modified Medical Research Council Dyspnoea Scale score ≥3.^5^ These criteria currently serve as a widely accepted reference for evaluating whether patients are candidates for lobectomy.

Postoperative locoregional recurrence has been reported more frequently after segmentectomy than after lobectomy.^1^ This observation highlights the importance of achieving adequate surgical margins and ensuring strict local control in sublobar resection. Similarly, in passive limited resection, local recurrence within the resected lobe constitutes a significant issue directly related to the surgical approach. In wedge resections, a surgical margin distance >10 mm has been associated with improved local control.^7^ Another widely adopted criterion is a surgical margin greater than or equal to the clinical tumour size or >20 mm, whichever is greater.^1^^,^^3^^,^^8^^,^^9^ In addition, the consolidation-to-tumour ratio (CTR) on preoperative computed tomography (CT), which reflects the proportion of the solid component within the tumour, is a well-established predictor of recurrence and prognosis following lung resection.^10–12^

Recent studies from Memorial Sloan Kettering Cancer Center have proposed refined models to assess surgical risk and guide treatment selection for stage I NSCLC.^13^^,^^14^ These frameworks incorporate frailty, pulmonary function (FEV_1_, DLCO), and prior thoracic surgery to distinguish candidates for minimally invasive surgery (MIS) versus stereotactic body radiotherapy (SBRT). One model identified poor performance status, reduced lung function, and prior resection as predictors of SBRT selection.^13^ Another study suggested lowering traditional postoperative thresholds (e.g., ppoFEV_1_ or ppoDLCO <60%) to 45% in the MIS era to better predict complications without excessive testing.^14^ These insights support evolving risk stratification in real-world practice.

Despite its clinical relevance, data on the outcomes of passive limited resection in high-risk patients remain limited,^5^^,^^6^^,^^15^ especially for tumours >20 mm.^5^ In real-world settings, the difficulty of securing adequate surgical margins in larger tumours may contribute to an increased risk of local recurrence. The aim of the present study was to evaluate real-world characteristics and outcomes of limited resection for NSCLC tumours >20 mm and to identify predictors of local recurrence.

## PATIENTS AND METHODS

### Study design and population

**Figure 1.**
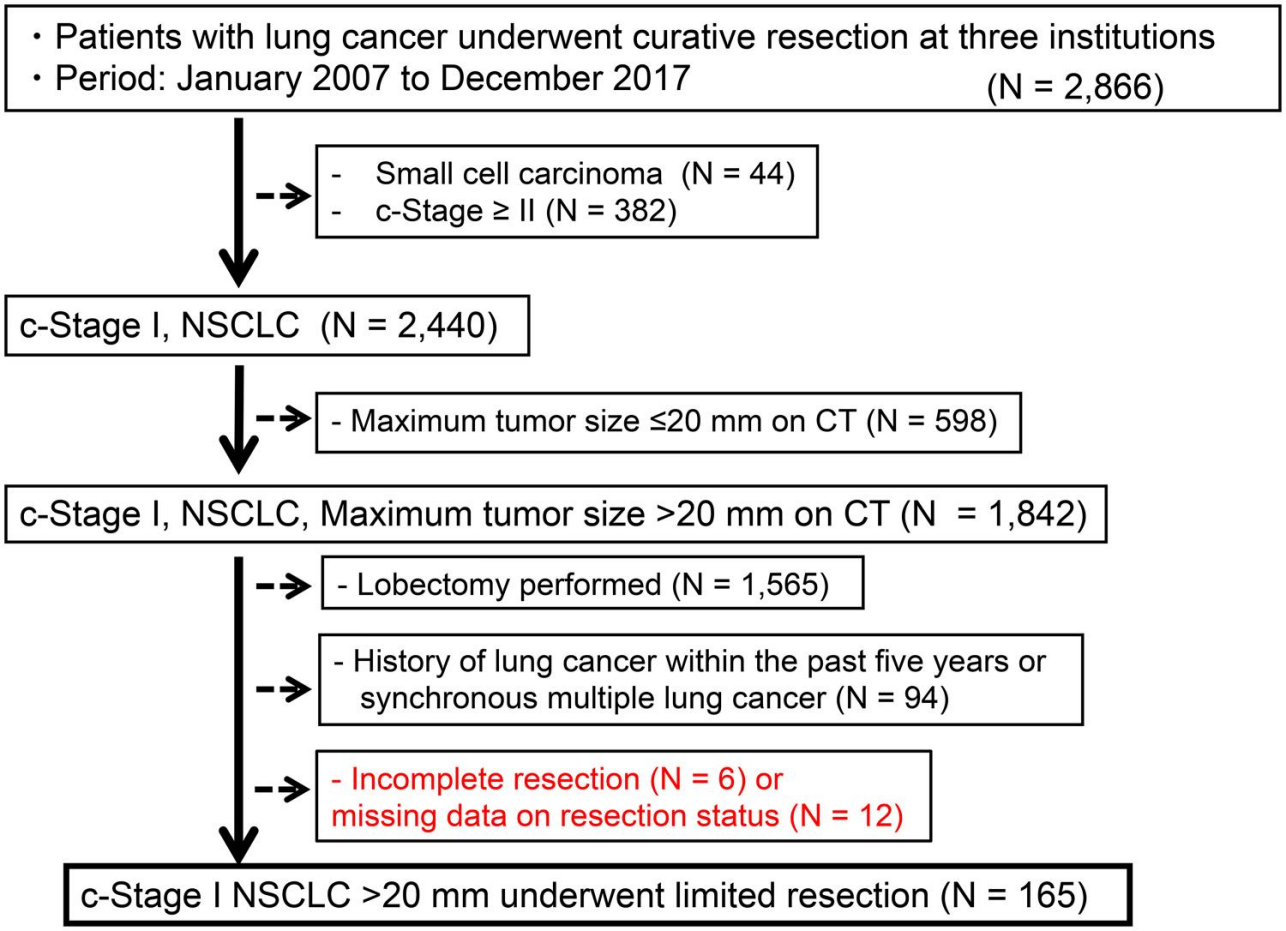
Flowchart Illustrating the Patient Selection Process. Among 2866 patients with c-Stage I NSCLC tumours >20 mm, 165 who underwent limited resection were included after applying exclusion criteria

This study was conducted in accordance with the Declaration of Helsinki and was approved by the Ethics Committee of Keio University School of Medicine (No. 20231021, approved on October 1, 2024) and affiliated institutions. The requirement for informed consent was waived due to the retrospective nature of the study. Between January 2007 and December 2017, a total of 2866 patients underwent surgical resection for primary lung cancer at 3 institutions affiliated with Keio University. Of these, 165 patients were included who were diagnosed with clinical stage IA NSCLC (cN0M0, 8th TNM edition), had a radiologic tumour >20 mm, and had undergone segmentectomy or wedge resection. In particular, nodal dissection was frequently omitted by the attending surgeon based on tumour characteristics, patient frailty, and lung function. Segmentectomy was favoured for centrally located tumours requiring larger margins, whereas wedge resection was selected for patients with severe interstitial changes or poor reserve. Exclusion criteria are detailed in **Figure 1**. During this period, the surgical approach at the 3 institutions was mainly video-assisted thoracoscopic surgery (VATS). Surgical margins were targeted at ≥ the tumour diameter or ≥2 cm when feasible. Complete (R0) resection was defined according to the International Association for the Study of Lung Cancer (IASLC) classification: tumour-free margins, systematic nodal dissection (≥3 mediastinal stations including station 7 and ≥3 hilar/parenchymal nodes), and negative pleural lavage cytology (PLC).^16^ PLC is not mandated in Japanese guidelines and was not performed in our cohort. In high-risk patients undergoing limited resection, mediastinal dissection was often omitted in sublobar resections.

### Data collection and follow-up

Clinical and pathological data, including perioperative outcomes and recurrence status, were collected from medical records. We followed up with patients every 3 months for the first 2 years postoperatively and every 6 months thereafter. Routine surveillance included chest-to-abdomen CT scans every 6 months. Additional imaging, such as brain magnetic resonance imaging or positron emission tomography, was performed when recurrence was suspected. When a solitary lung nodule was identified postoperatively, biopsy was conducted where feasible. Lesions were classified based on the Martini and Melamed criteria.^17^ Locoregional recurrence was defined as recurrence within the ipsilateral hemithorax, and local recurrence was defined as recurrence within the resected lobe.^5^ The OS and disease-free survival (DFS) were analysed as time-to-event outcomes, and observations were censored at the final follow-up or upon death. Exploratory subgroup comparisons between segmentectomy and wedge resection were performed to describe procedural characteristics and outcomes for reference purposes (**Table S1**).

### Radiological and pathological assessment

The CTR was calculated by dividing the maximum diameter of the solid component of the tumour by the total size on lung-window CT images with 1-2 mm slice thickness. Tumours were categorized as ground-glass opacity (GGO)-predominant (CTR ≤ 0.5) or solid-predominant (CTR > 0.5). The surgical margin was defined as the shortest distance between the tumour edge and the visceral pleura-free cut end. These distances were reassessed microscopically by pathologists at each institution using formalin-fixed resected specimens.

### Statistical analysis

Categorical variables were summarized using frequencies and percentages; medians and interquartile ranges were used for continuous variables. The OS and DFS were estimated using Kaplan-Meier curves; prognostic factors were analysed with Cox proportional hazards models. Variables that were significant in univariable analysis were included in multivariable models through a stepwise selection method (entry, *P* < .05; removal, *P* > .10); however, those that were deemed clinically unrelated to outcomes or those for which hazard ratios (HR) could not be estimated were excluded. To estimate time to recurrence in the presence of competing risks, cumulative incidence functions were used, with death treated as a competing event. Multivariable analysis of recurrence was performed using the Fine-Gray subdistribution hazard model.

The primary end-point was local recurrence. Locoregional recurrence was also evaluated as a reference value. We used competing risk analysis to estimate the cumulative incidence of local recurrence. Other types of recurrence and death were included as competing events. The Fine-Gray subdistribution hazard model was used to identify predictors of local recurrence. All analyses were performed using EZR (Saitama Medical Center, Jichi Medical University, Saitama, Japan), a graphical user Áinterface for R (The R Foundation for Statistical Computing, Vienna, Austria).

## RESULTS

### Clinicopathological characteristics and perioperative outcomes

Among the 165 patients, 146 (88.5%) underwent passive limited resection based on clinically documented reasons indicating elevated surgical risk for lobectomy. In routine clinical practice, patients were judged to be “high-risk” by their respective attending physicians when deemed unsuitable for lobectomy due to comorbidities, limited pulmonary function, advanced age, or other clinical considerations, and thus received sublobar resection. The specific reasons for limited resection are summarized in **Table S2**. In the remaining 19 patients (11.5%), no explicit rationale for limited resection was documented, highlighting the inherent heterogeneity of real-world surgical decision-making in this population. By IASLC criteria, all cases were classified as R [uncertain (un)], primarily due to omission or limitation of mediastinal lymph node dissection and the absence of PLC.

The clinical and pathological features of the patients are shown in Table 1. The median tumour size was 25 mm (interquartile range 22.0-30.0 mm); 70% of tumours measured 20-30 mm, and 30% were >30 mm. On preoperative CT, 40 tumours (24.2%) were GGO-predominant and 125 (75.8%) were solid-predominant, with 90 that were pure-solid (CTR = 1.0) (54.5%). Segmentectomy and wedge resection were performed in 97 (58.8%) and 68 patients (41.2%), respectively. Procedural characteristics and perioperative outcomes stratified by surgical approach are summarized in **Table S1** as an exploratory analysis. Lymph node dissection was performed in all segmentectomies but omitted in 54 (55.7%) wedge resections. The median margin distance was 11 mm. Postoperative complications occurred in 31 patients (8.8%): 18 (10.9%) experienced Common Terminology Criteria for Adverse Events grade 1-2 events (e.g., dyspnoea, atelectasis, wound infection, anaemia) and 13 (7.9%) experienced grade 3 events (e.g., arrhythmia, empyema, cerebral infarction, prolonged air leak). No 30- or 90-day mortalities occurred. Pathological findings showed that 117 (70.9%) patients had adenocarcinoma; 49 (29.7%) had visceral pleural invasion (VPI), and 5 (3.0%) had lymph node metastasis.

**Table 1.** Patient and Tumour Characteristics

Variables	Overall (*N* = 165)
Age, years	74 (67-78)
Sex	
Male	88 (53.3)
Female	77 (46.7)
Clinical tumour size, mm	25.0 (22.0-30.0)
Size distribution	
≥2 cm, <3 cm	116 (70.3)
≥3 cm, <4 cm	40 (24.2)
≥4 cm, <5 cm	9 (5.5)
Solid-part size, mm	22.0 (17.0-25.0)
CTR	1.0 (0.5-1.0)
Predominant pattern	
GGO-predominant	40 (24.2)
Solid-predominant	125 (75.8)
Pure-solid tumour (CTR = 1.0)	90 (54.5)
Tumour localization	
Central	17 (10.3)
Peripheral	148 (89.7)
PET, SUV max	4.0 (1.8-8.3)
Missing case	44 (26.7)
Pulmonary function	
%FEV1.0	73.0 (66.4-86.0)
Surgical procedure	
Segmentectomy	97 (58.8)
Wedge resection	68 (41.2)
Surgical approach	
Open	6 (3.6)
VATS	159 (96.4)
Margin distance, mm	11 (6-18)
Lymph node dissection	111 (67.3)
Adverse event (complication)	31 (18.8)
Grade 1-2	18 (10.9)
Grade ≥3	13 (7.9)
Cardio-pulmonary complication	15 (9.1)
30-, 90-day mortality	0
Re-operation	0
Length of stay, day	10.0 (8.0-12.0)
Lymph node dissection	111 (67.3)
Histology	
Adenocarcinoma	117 (70.9)
Non-adenocarcinoma	48 (29.1)
Pathological tumour size, mm	25.0 (21.0-30.0)
Invasive part size, mm	22.0 (13.0-28.0)
Lymph node metastasis	5 (3.0)
Lymphatic invasion	40 (24.2)
Vascular invasion	38 (23.0)
Visceral pleural invasion	49 (29.7)
STAS	42 (25.5)
Adjuvant chemotherapy	10 (6.1)
Site of first postoperative recurrence	
Overall	38 (23.0)
Distant	11 (6.7)
Locoregional	27 (16.4)
Local	13 (7.9)
Others	14 (8.5)

Values are listed as *n* (%) or median (interquartile range).

Abbreviations: %FEV1,% predicted forced expiratory volume in 1 second; CTR, consolidation-to-tumour ratio; GGO, ground-glass opacity; PET, positron emission tomography; STAS, spreading through the air space; SUV, standard uptake value; VATS, video-assisted thoracoscopic surgery.

During a median follow-up time of 60 months (interquartile range, 30-85 months), 38 of 165 patients (23.0%) experienced recurrence, of which 11 (6.7%) were distant and 27 (16.4%) were locoregional recurrences (**Table S3**). Thirteen (7.9%) of the locoregional recurrences were classified as local, 3 (1.8%) were ipsilateral lobe metastases, 7 (4.2%) were nodal, and 4 (2.4%) were pleural dissemination.

### Survival outcomes

**Figure 2.**
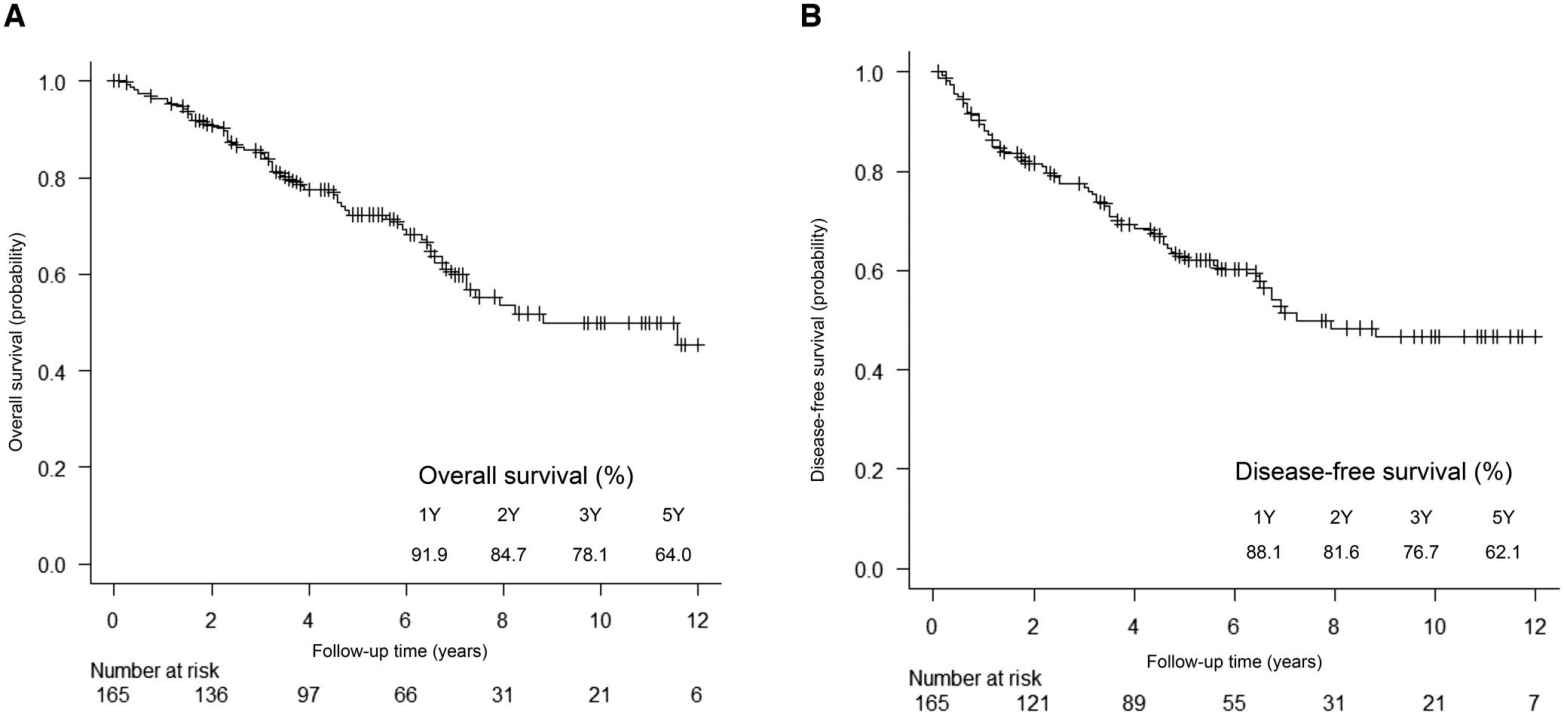
Kaplan-Meier Survival Curves. (A) Overall survival and (B) disease-free survival of patients with c-Stage I NSCLC tumours >20 mm who underwent passive limited resection

A total of 165 patients with 13 local recurrence events were included in the study. Kaplan-Meier survival curves are shown in **Figure 2** (A: OS, B: DFS). The 2- and 5-year OS rates were 84.7% and 64.0%, respectively, and the DFS rates were 81.6% and 62.1%. Cox regression identified independent predictors of DFS as: predominant pattern on CT (HR, 2.65; 95% CI, 1.08-7.95; *P* = .05), adenocarcinoma histology (HR, 2.82; 95% CI, 1.73-4.60; *P* < .001), and VPI (HR, 3.51; 95% CI, 2.16-5.71; *P* < .001) (**Table 2**; **Figure S1**). As a supplementary analysis, we also compared outcomes between the sublobar resection cohort (*n* = 165) and a reference lobectomy cohort (*n* = 1397) selected using similar radiological criteria. Although OS and DFS were significantly better in the lobectomy group (*P* < .001), rates of postoperative recurrence (20.1% vs 23.0%, *P* = .41) and grade ≥3 adverse events (15.8% vs 18.8%, *P* = .32) were not significantly different (**Figure S2**; **Table S4**). To evaluate whether treatment period influenced outcomes, we compared recurrence and survival rates, between 2 calendar cohorts: 2007-2012 and 2013-2017. No significant differences were observed between the 2 groups (**Table S5**).

**Table 2.** Univariable and Multivariable Analyses to Estimate the Predictors of Disease-Free Survival

Variables		Univariable		Multivariable	
		HR (95% CI)	*P*-value	Adjusted HR (95% CI)	*P*-value
Clinical tumour size, mm	Continuous	1.01 (0.97-1.04)	.74		
Solid-part size, mm	Continuous	1.04 (1.02-1.07)	<.001		
Predominant pattern	Solid-predominant	5.55 (2.46-15.89)	<.001	2.65 (1.08-7.95)	.05
Pure-solid tumour	Yes	2.63 (1.55-4.49)	<.001		
Margin distance, mm	Continuous	1.01 (0.99-1.04)	.21		
	<10 mm	1.08 (0.66-1.77)	.77		
	<20 mm	1.34 (0.78-2.30)	.29		
	<Whole tumour size	1.46 (0.69-3.06)	.32		
Surgical procedure	Wedge resection	1.63 (1.0-2.66)	.05		
Adjuvant chemotherapy	Yes	0.59 (0.18-1.87)	.37		
Tumour histology	Adenocarcinoma	3.08 (1.90-4.99)	<.001	2.82 (1.73-4.60)	<.001
Pathological tumour size, mm	Continuous	1.03 (1.01-1.06)	.01		
Invasive part size, mm	Continuous	1.03 (1.02-1.05)	.004		
Lymph node metastasis	Positive	3.36 (1.03-10.99)	.05		
Lymphatic invasion	Positive	2.59 (1.56-4.23)	<.001		
Vascular invasion	Positive	1.69 (0.98-2.80)	.06		
Visceral pleural invasion	Positive	3.75 (2.32-6.11)	<.001	3.51 (2.16-5.71)	<.001
STAS	Positive	1.97 (1.19-3.21)	.009		

Abbreviations: CI, confidence interval; HR, hazard ratio; STAS, spread through air space.

### Cumulative incidence of recurrence

**Figure 3.**
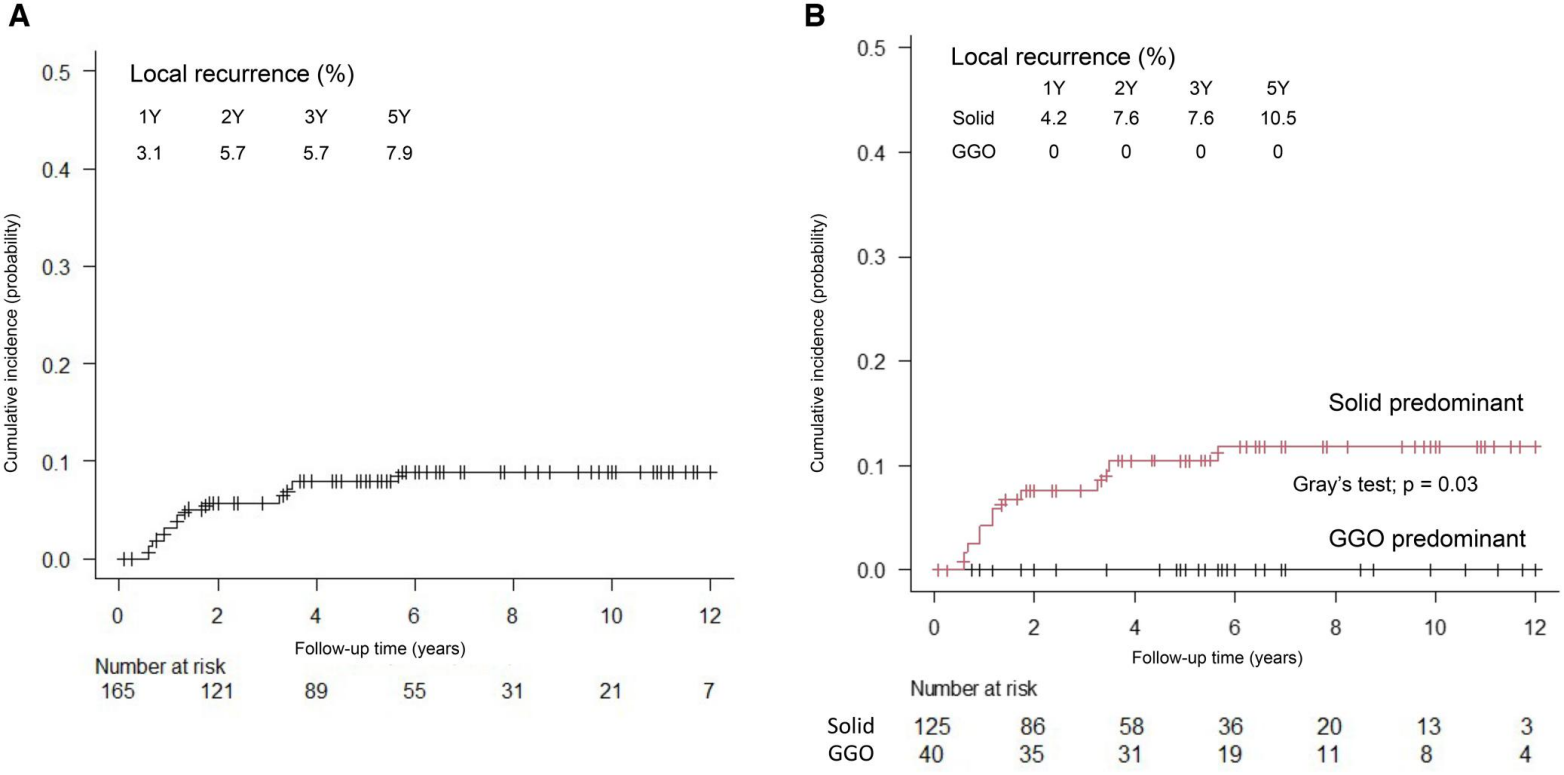
Cumulative Incidence of Local Recurrence. Cumulative incidence curves of local recurrence overall (A) and stratified by predominant pattern on CT (B: ground-glass opacity-predominant vs. solid-predominant). Gray’s test indicated a statistically significant difference (*P* = .03). Abbreviation: GGO, ground-glass opacity

**Figure 3A** shows the cumulative incidences of local recurrence, which were 3.1%, 5.7%, and 7.9% at 1, 2, and 5 years, respectively. In univariable competing risk analysis (**Table 3**), predominant pattern on CT, surgical procedure, lymph node metastasis, lymphatic and vascular invasion, VPI, and margin distance < 10 mm were significantly associated with local recurrence. In multivariable Fine-Gray analysis (**Table 3**), the only independent predictor was predominant pattern on CT (HR, ∞ [approaching infinity], *P* < .001). No local recurrence was observed in GGO-predominant tumours (**Figure 3B**; **Table S6**). Cumulative incidences of locoregional recurrence were 10.3%, 17.7%, and 32.6% at 1, 2, and 5 years, respectively (**Figure S4A**). Multivariable analysis identified predominant pattern on CT (HR, ∞, *P* < .001) and VPI (HR, 3.43; 95% CI, 1.57-7.52; *P* = .002) as independent predictors of locoregional recurrence (**Table S7**; **Figures S4B and S4C**). No recurrence was observed in the GGO-predominant group, and pure-solid tumours were not independently associated with recurrence.

**Table 3.** Univariable and Multivariable Analysis to Estimate the Predictor of Early Local Recurrence in the Same Lobe With Fine-Gray Competing Risk Analysis

Variables		Univariable		Multivariable	
		HR (95% CI)	*P*-value	Adjusted HR (95% CI)	*P*-value
Clinical tumour size, mm	Continuous	0.97 (0.91-1.04)	.43		
Solid-part size, mm	Continuous	1.02 (0.98-1.06)	.36		
Predominant pattern	Solid-predominant	∞	<.001	∞	<.001
Pure-solid tumour	Yes	1.90 (0.59-6.12)	.28		
Margin distance, mm	Continuous	0.94 (0.85-1.05)	.28		
	<10 mm	3.65 (1.14-11.7)	.03		
	<20 mm	3.63 (0.48-27.4)	.21		
	<Whole tumour size	1.28 (0.17-9.52)	.81		
Surgical procedure	Wedge resection	3.79 (1.20-12.01)	.02		
Tumour histology	Adenocarcinoma	1.62 (0.53-4.92)	.40		
Pathological tumour size, mm	Continuous	0.99 (0.95-1.03)	.64		
Invasive part size, mm	Continuous	1.01 (0.97-1.05)	.59		
Lymph node metastasis	Positive	N.A.	.001		
Lymphatic invasion	Positive	5.63 (1.87-16.9)	.002		
Vascular invasion	Positive	3.33 (1.12-9.85)	.03		
Visceral pleural invasion	Positive	5.57 (1.75-17.7)	.004		
STAS	Positive	2.53 (0.86-7.43)	.09		

Abbreviations: CI, confidence interval; HR, hazard ratio; STAS, spread through air space.

## DISCUSSION

This study retrospectively investigated real-world characteristics and outcomes of limited resection for clinical stage I NSCLC tumours >20 mm. The results revealed that most cases were passive limited resections. Our study reported Common Terminology Criteria for Adverse Events grade ≥3 adverse events in 8% of patients, with no perioperative mortality. This was notably lower than in the ACOSOG Z4032 trial, in which grade ≥3 events occurred in 30% of patients, and 30- and 90-day mortality rates were 0.9% and 2.3%, respectively.^5^ Differences in perioperative management, surgical techniques (VATS in the present study vs 36% open surgery in the ACOSOG Z4032 trial), and study periods might have been responsible for this discrepancy. Nonetheless, the consistently low mortality observed in both studies supports the viability of sublobar resection, even in predominantly high-risk populations.

While prior studies emphasized surgical margins,^1^^,^^5^ emerging evidence suggests tumour biology may more strongly influence local recurrence. In our cohort, shorter margin distances were associated with recurrence in univariable analysis but not in multivariable analysis. This is consistent with Fick *et al.*, who identified ≥3 high-risk features—such as high SUVmax, sublobar resection, higher IASLC grade, lymphovascular and pleural invasion, and tumour size—as independent predictors of recurrence in stage I NSCLC.^18^ Notably, histological grade and metabolic activity were stronger predictors than surgical technique or margin length. Our study could not assess variables like IASLC grade due to limitations in retrospective pathology. However, the significant association between solid-predominant tumours and recurrence supports the idea that intrinsic tumour characteristics may outweigh surgical factors in determining prognosis. Accordingly, future studies should prioritize biologically based risk stratification over margin length, particularly in high-risk or borderline-operable patients. The role of SBRT also merits consideration. Recent studies have proposed refined criteria for selecting SBRT over surgery based on frailty and lung function.^13^^,^^14^ Comparative studies of sublobar resection and SBRT in these populations are warranted.

The results of this study indicated that predominant pattern on CT strongly predicted both local and locoregional recurrence. Supplementary analysis revealed that patients with GGO-predominant tumours experienced no local recurrence, even with surgical margin distances less than the total tumour size or <20 mm (**Table S8**). Conversely, solid-predominant tumours recurred despite having margins greater than or equal to the total tumour size. The Japan Clinical Oncology Group 1211 trial similarly demonstrated a minimal risk of local recurrence following segmentectomy for GGO-predominant tumours measuring ≤30 mm, including those 20-30 mm in size.^4^ These findings suggest that local recurrence could potentially be prevented in GGO-predominant NSCLC if complete resection is achieved, whereas solid-predominant tumours may necessitate more cautious surgical planning.

In the Japanese Joint Committee of Lung Cancer Registry (*n* = 18 973), the 5-year disease-free survival (DFS) rates were 74.8% for stage IA3 and 71.5% for stage IB, with grade ≥3 complications in 8.3% and a 90-day mortality rate of 1.26%.^19^ In our cohort of patients deemed unfit for standard surgery, the 5-year DFS was 62.1%, grade ≥3 complications occurred in 7.9%, and no perioperative deaths were observed. We also conducted a supplementary analysis comparing our sublobar cohort with a historical lobectomy cohort. Although DFS was significantly better in the lobectomy group—likely reflecting differences in baseline fitness—rates of recurrence and major complications were comparable. These findings suggest that limited resection for NSCLC >20 mm can achieve acceptable oncologic and perioperative outcomes comparable to lobectomy in appropriately selected high-risk patients.

### Study limitations

This study has several limitations. First, its retrospective observational design precludes causal inference and introduces potential selection bias. Second, surgical indications were not standardized across institutions, and “high-risk” status was defined variably by attending surgeons. Some patients lacked documentation clarifying the rationale for limited resection, reducing internal validity and generalizability. Third, the number of local recurrence events (*n* = 13) was small, limiting the statistical power to detect associations and causing convergence issues in multivariable Fine-Gray models. A post hoc power analysis suggested that at least 34 events would be required to detect a hazard ratio of 0.4 with 80% power. Therefore, the results of multivariable analyses should be interpreted with caution and considered exploratory. Fourth, important biological factors such as IASLC grade were not consistently available across institutions, limiting comprehensive assessment of tumour biology. Fifth, although the study included 3 affiliated institutions, inter-institutional differences in imaging interpretation, surgical margin evaluation, and nodal dissection may limit reproducibility. In particular, nodal dissection was often omitted in wedge resections, potentially affecting recurrence assessment. However, a sensitivity analysis of patients who underwent lymph node dissection (*N* = 111) yielded results consistent with the overall cohort (**Table S9**). Lastly, variation in pathological subtypes and methods of CTR and margin assessment may further affect interpretation. Despite these limitations, our findings suggest that preoperative CT features—particularly a solid-predominant pattern—may be more informative for recurrence risk than resection type alone. Prospective studies using standardized criteria are needed to validate these observations.

## CONCLUSIONS

In the selected patients, limited resection for NSCLC tumours >20 mm was associated with acceptable perioperative and oncologic outcomes, particularly in tumours with a GGO-predominant pattern. These findings suggest that surgical management and follow-up strategies may need to be adjusted according to the predominant radiological features observed on preoperative CT imaging. They also underscore the need to define clear criteria for selecting the optimal treatment modality—lobectomy, sublobar resection, or SBRT—for each patient.

## Supplementary Material

ezaf322_Supplementary_Data

## Data Availability

Data available on request.
