# Supplementary material for: Real-World Outcomes of Limited Resection for Tumours Greater Than 20 mm in Non-Small Cell Lung Cancer
Source: Eur J Cardiothorac Surg. 2025 Sep 29;67(10):ezaf322. doi: 10.1093/ejcts/ezaf322 (PMC12500328; doi:10.1093/ejcts/ezaf322)

Spp Fig.1 Kaplan–Meier curves for DFS

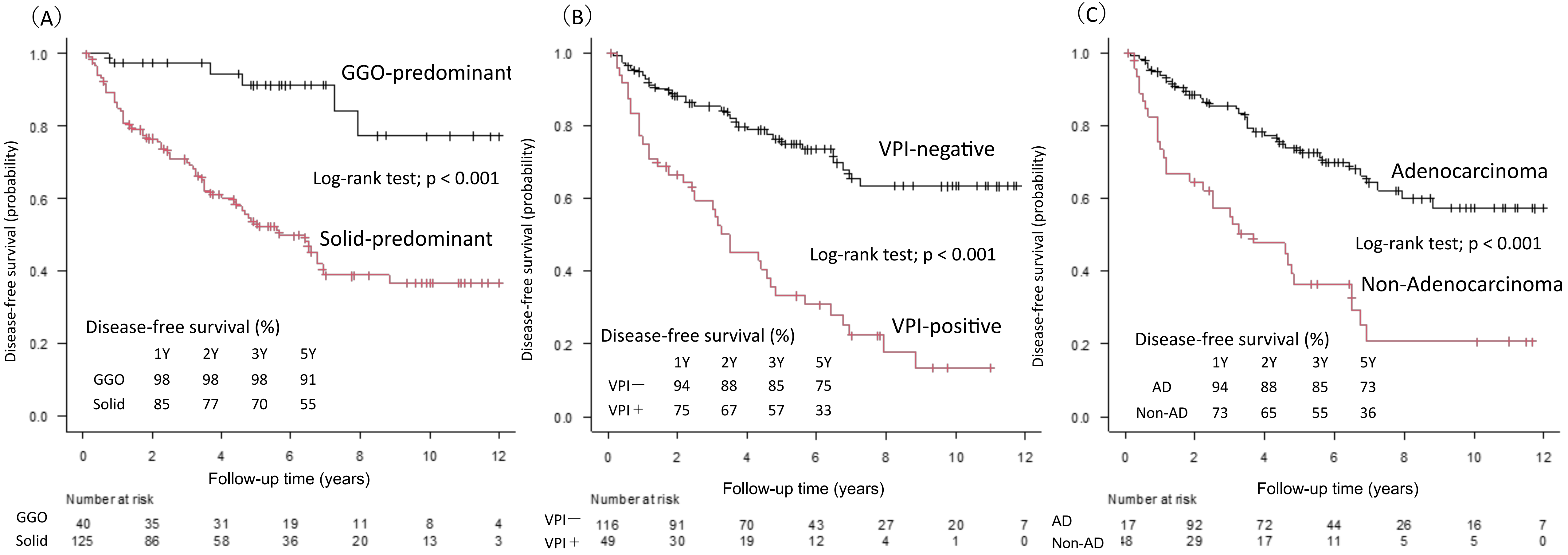

Spp Fig.2 Kaplan–Meier survival curves comparing overall survival (A) and disease-free survival (B) between the sublobar resection and the reference lobectomy cohort

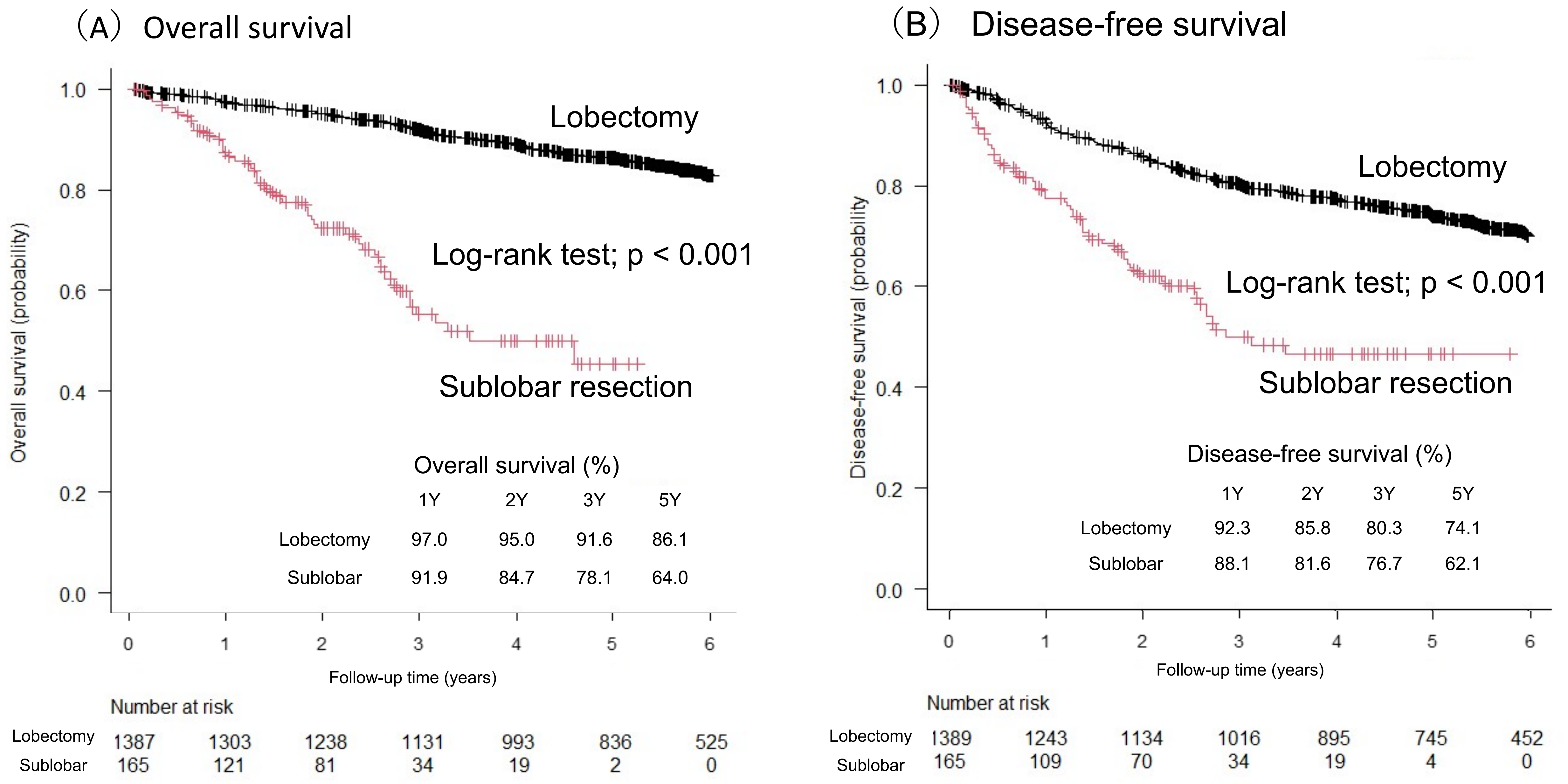

Spp Fig.3 Cumulative incidence of locoregional recurrence

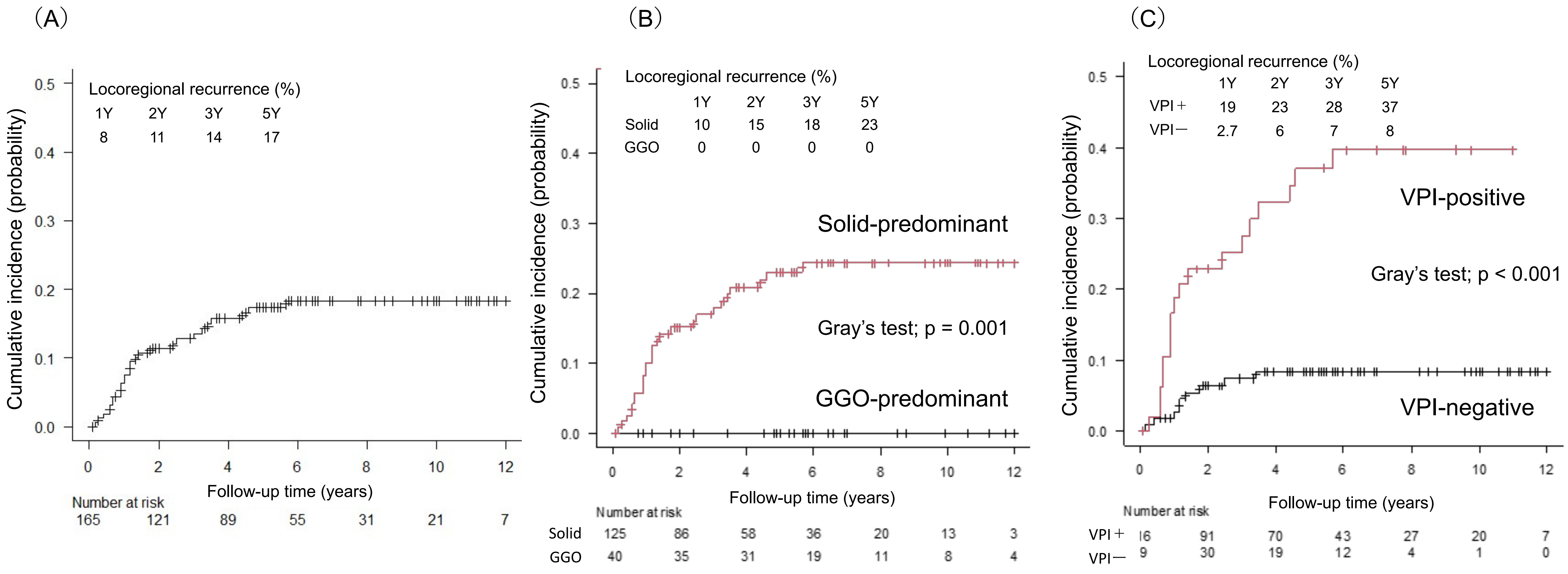

Spp Fig.4　Disease-free survival and cumulative incidence of recurrence according to surgical procedure

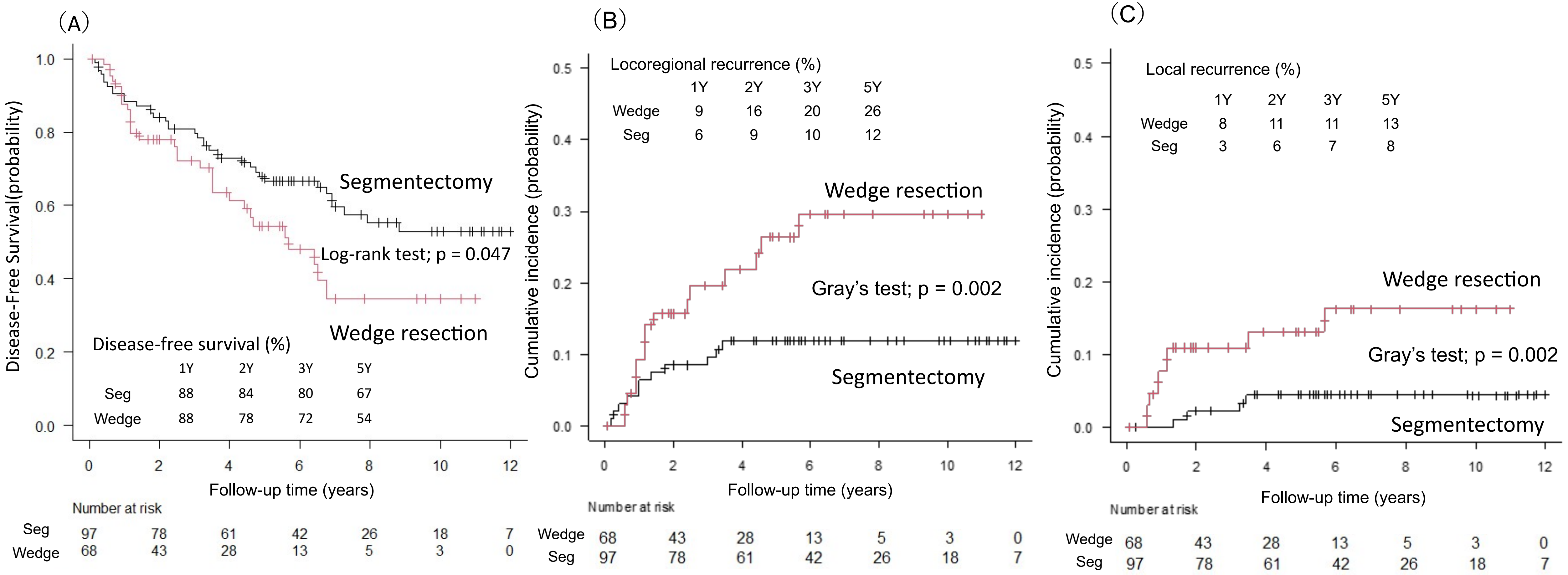

Supplement: ezaf322_Supplementary_Data [file ezaf322_supplementary_data.zip › Supplementary Figure revision.pdf]
