# Supplementary material for: Real-World Outcomes of Limited Resection for Tumours Greater Than 20 mm in Non-Small Cell Lung Cancer
Source: Eur J Cardiothorac Surg. 2025 Sep 29;67(10):ezaf322. doi: 10.1093/ejcts/ezaf322 (PMC12500328; doi:10.1093/ejcts/ezaf322)
Supplement: ezaf322_Supplementary_Data [file ezaf322_supplementary_data.zip › Spp_tables_revision.docx]

**Supplementary Table S1**. Comparison of patient and tumor characteristics, perioperative outcomes and recurrence patterns between according to surgical procedure.

| Variables | Segmentectomy  (N = 97) | Wedge resection  (N = 68) | p-value |
| --- | --- | --- | --- |
| Age, years | 70 (65–76) | 76 (72–79) | <0.001 |
| Sex |  |  |  |
| Male | 64 (66.7) | 51 (75.0) | 0.23 |
| Female | 33 (34.0) | 17 (25.0) |  |
| Clinical tumor size, mm | 26.0 (23.0–31.0) | 25 .0 (21.7–28.0) | 0.015 |
| Distribution |  |  |  |
| ≥2 cm, <3 cm | 62 (63.9) | 54 (79.4) | 0.09 |
| ≥3 cm, <4 cm | 29 (29.9) | 11 (16.2) |  |
| ≥4 cm, <5 cm | 6 (6.2) | 3 (4.4) |  |
| Solid-part size, mm | 22.0 (15.0–26.0) | 21.5 (20.0–25.0) | 0.62 |
| CTR |  |  |  |
| Pure-solid tumor (CTR=1.0) | 48 (49.5) | 42 (61.8) | 0.15 |
| Predominant pattern |  |  | 0.14 |
| Solid-predominant | 69 (71.1) | 56 (82.4) |  |
| GGO-predominant | 28 (28.9) | 12 (17.6) |  |
| Tumor localization |  |  | 0.51 |
| Central | 13 (13.4) | 12 (17.6) |  |
| Peripheral | 84 (86.6) | 56 (82.4) |  |
| PET, SUV max | 3.4 (1.8–7.3) | 4.7 (2.3–8.4) | 0.20 |
| Missing | 65 (67.0) | 55 (80.9) |  |
| Margin distance, mm | 13 (8–20) | 10 (5–14) | 0.001 |
| Adverse event | 21 (21.6) | 10 (14.7) | 0.31 |
| Grade 1–2 | 14 (14.4) | 4 (5.9) |  |
| Grade ≥3 | 7 (7.2) | 6 (8.8) |  |
| 30-, 90-day mortality | 0 | 0 | ― |
| Lymph node dissection | 97 (100) | 14 (20.5) | 0.93 |
| Histology |  |  | 0.86 |
| Adenocarcinoma | 68 (70.1) | 49 (72.1) |  |
| Non-adenocarcinoma | 29 (29.9) | 19 (27.9) |  |
| Pathological tumor size, mm | 25.0 (22.0–32.0) | 24.5 (20.8–27.3) | 0.04 |
| Invasive part size, mm | 22.0 (10.0–28.0) | 22.0 (17.8–27.0) | 0.49 |
| Lymph node metastasis | 4 (4.1) | 1 (1.5) | 0.50 |
| Lymphatic invasion | 16 (16.5) | 24 (35.3) | 0.009 |
| Vascular invasion | 19 (19.6) | 19 (27.9) | 0.26 |
| Visceral pleural invasion | 19 (19.6) | 30 (44.1) | 0.001 |
| STAS | 22 (22.7) | 20 (29.4) | 0.37 |
| Adjuvant chemotherapy | 9 (9.3) | 1 (1.5) | 0.05 |
| First recurrent site after surgery |  |  |  |
| Overall | 20 (20.6) | 18 (26.5) | 0.45 |
| Distant | 11 (11.3) | 5 (7.4) | 0.43 |
| Locoregional | 13 (13.4) | 9 (13.2) | 1 |
| Local | 4 (4.1) | 9 (13.2) | 0.04 |

Values are listed as n (%) or median (interquartile range).

Variables were compared using Fisher’s exact test for categorical outcomes and t tests for continuous variables.

CTR, consolidation/tumor ratio; GGO, ground glass opacity; PET, positron emission tomography; STAS, spreading through the air space; SUV, standard uptake value.
Note: These exploratory analyses are descriptive only and not adjusted for baseline differences.

**Supplementary Table S2.** Primary indications for limited resection

| Reasons | The number of patients  (N = 165) | Values |
| --- | --- | --- |
| Advanced age | 9 (5.5) | median 84.0 (84.0-87.0) |
| Comorbidity | 116 (70.3) | － |
| Advanced malignancies in other organs | 69 (41.8) | － |
| Heart disease | 34 (20.6) | － |
| Steroid-treated interstitial pneumonia | 4 (2.4) | － |
| Cerebrovascular disease | 7 (4.2) | － |
| Renal failure | 2 (1.2) | － |
| Poor pulmonary function　(%FEV1.0) | 17 (10.3) | median 45.0 (37.4-46.0) |
| Unclear | 19 (11.5) | － |

Number of patients and values are listed as n (%) and median (interquartile range), respectively.

%FEV1, Percent predicted forced expiratory volume in one second.

**Supplementary Table S3.** First recurrence site after surgery

| Recurrence site | Total  (N = 165) |
| --- | --- |
|  |  |
| **Overall** | 38 (23) |
| **Distant** | 11 (7) |
| Contralateral lung | 2 (1) |
| Contralateral lymph node | 1 (1) |
| Bone | 4 (2) |
| Brain | 2 (1) |
| Liver | 2 (1) |
| **Locoregional** | 27 (16) |
| Local | 13 (8) |
| Others | 14 (8) |
| Lung (other lobes) | 3 (2) |
| Lymph node | 7 (4) |
| Pleural dissemination | 4 (2) |

Clinical values are listed as n (%).

**Supplementary Table S4.** Comparison of Clinical Outcomes Between Sublobar Resection and Reference Lobectomy Cohorts.

| Outcome | Lobectomy  (N = 1397) | Sublobar resection  (N = 165) |
| --- | --- | --- |
| Postoperative recurrence | 281 (20.1%) | 38 (23.0%) |
| Perioperative adverse events | 221 (15.8%) | 31 (18.8%) |

Clinical values are listed as n (%).

Perioperative adverse events were categorized according to the Common Terminology Criteria for Adverse Events (CTCAE), all grades.

Note: These exploratory analyses are descriptive only and not adjusted for baseline differences.

**Supplementary Table S5.** Comparison of Clinicopathological Characteristics and Outcomes Between Patients Treated in the Early and Late Study Periods

| Characteristics/ Outcomes | Study period | | p-value |
| --- | --- | --- | --- |
|  | 2007-2012  (N = 122) | 2013-2017  (N = 43) |  |
| Surgical procedure |  |  |  |
| Segmentectomy | 75 (62) | 19 (44) | 0.07 |
| Wedge resection | 47 (38) | 24 (56) |  |
| Lymph node dissection | 82 (67) | 19 (44) | 0.20 |
| Recurrence | 29 (24) | 5 (12) | 0.12 |
| Overall survival |  |  |  |
| Median | NA | NA | 0.76 |
| 2-year (%) | 94.2 | 89.1 |  |
| 5-year (%) | 79.5 | 72.3 |  |
| Disease free survival |  |  |  |
| Madian | NA | NA | 0.88 |
| 2-year (%) | 88.6 | 81.5 |  |
| 5-year (%) | 71.3 | 69.3 |  |
| Observation period (month) | 89.5 (46.0, 200.8) | 41.0 (21.5, 110.5) | <0.001 |

Clinical values are listed as n (%) or median (interquartile range).

Variables were compared using the Fisher’s exact test for categorical outcomes, t-tests for continuous variables and log-rank test for time-to-event outcomes.

NA, Not applicable

**Supplementary Table S6.** Patient and tumor characteristics according to Solid predominance

| Characteristics | GGO-predominant  (N = 40) | Solid-predominant  (N = 125) | p-value |
| --- | --- | --- | --- |
| Age, years | 74.5 (52–90) | 74 (45–89) | 0.86 |
| Sex |  |  |  |
| Male | 18 (45) | 70 (56) | 0.17 |
| Female | 22 (55) | 55 (44) |  |
| Clinical tumor size, mm | 25 (21–49) | 25 (21–49) | 0.83 |
| Distribution |  |  |  |
| ≥2 cm, <3 cm | 29 (73) | 87 (70) | 0.95 |
| ≥3 cm, <4 cm | 9 (23) | 31 (25) |  |
| ≥4 cm, <5 cm | 2 (4) | 7 (5) |  |
| Solid-part size, mm | 9 (0–22) | 24 (16–49) | <0.001 |
| CTR | 0.29（0–0.50） | 1.0 (0.53–1.0) | <0.001 |
| Tumor localization |  |  |  |
| Central | 5 (13) | 12 (10) | 0.80 |
| Peripheral | 35 (87) | 113 (90) |  |
| PET, SUV max | 1.3 (0–5.5) | 5.3 (0–18.6) | <0.001 |
| Missing | 10 (25) | 34 (27) |  |
| Surgical procedure |  |  |  |
| Segmentectomy | 28 (70) | 69 (55) | 0.14 |
| Wedge resection | 12 (30) | 56 (45) |  |
| Margin distance, mm | 13 (1–52) | 11 (1–50) | 0.92 |
| Lymph node dissection | 34 (85) | 77 (62) | 0.006 |
| Histology |  |  | <0.001 |
| Adenocarcinoma | 40 (100) | 77 (62) |  |
| Non-adenocarcinoma | 0 | 48 (38) |  |
| Pathological tumor size, mm | 24 (9–45) | 25 (14–60) | 0.02 |
| Invasive part size, mm | 4 (0–40) | 25 (8–60) | <0.001 |
| Lymph node metastasis | 0（0） | 5 (7) | 0.54 |
| Lymphatic invasion | 1（3） | 41 (33) | <0.001 |
| Vascular invasion | 0 (0) | 38 (30) | <0.001 |
| Visceral pleural invasion | 1 (3) | 48 (38) | <0.001 |
| STAS | 2 (5) | 40 (32) | <0.001 |
| First recurrent site |  |  |  |
| Overall | 0 | 38 (30) | <0.001 |
| Distant | 0 | 11 (9) |  |
| Locoregional | 0 | 27 (21) |  |

Clinical values are listed as n (%) or median (interquartile range).

Variables were compared using the Fisher’s exact test for categorical outcomes and t-tests for continuous variables.

CTR, consolidation/tumor ratio; GGO, ground glass opacity; PET, positron emission tomography; STAS, spread through air space; SUV, standardized uptake value.

**Supplementary Table S7.** Univariate and multivariate analysis to estimate the predictor of early locoregional recurrence with Fine–Gray competing risk analysis.

| Variables | | Univariate analysis | | Multivariate analysis | | |
| --- | --- | --- | --- | --- | --- | --- |
|  |  | HR  (95% CI) | p-value |  | Adjusted HR (95% CI) | p-value |
| Clinical tumor size, mm | Continuous | 0.99  (0.93–1.05) | 0.67 |  |  |  |
| Solid-part size, mm | Continuous | 1.04  (1.01–1.07) | 0.005 |  |  |  |
| Predominant pattern | Solid-predominant | ∞ | 0.004 |  | ∞ | <0.001 |
| Pure-solid tumor | Yes | 2.30  (0.91–5.83) | 0.08 |  |  |  |
| Margin distance, mm | Continuous | 1.01  (0.96–1.05) | 0.79 |  |  |  |
|  | ≤10 mm | 1.82  (0.39–1.73) | 0.6 |  |  |  |
|  | ≤20 mm | 1.03  (0.38–2.81) | 0.95 |  |  |  |
|  | ≤Whole tumor size | 1.19  (0.37–3.82) | 0.77 |  |  |  |
| Surgical procedure | Wedge resection | 1.97  (0.85–4.58) | 0.12 |  |  |  |
| Tumor histology | Adenocarcinoma | 2.60  (1.23–5.50) | 0.01 |  |  |  |
| Pathological tumor size, mm | Continuous | 1.02  (0.98–1.05) | 0.35 |  |  |  |
| Invasive part size, mm | Continuous | 1.03  (1.0–1.05) | 0.03 |  |  |  |
| Lymph node metastasis | Positive | N.A. | <0.001 |  |  |  |
| Lymphatic invasion | Positive | 4.01  (1.90–8.45) | <0.001 |  |  |  |
| Vascular invasion | Positive | 2.35  (1.07–5.12) | 0.03 |  |  |  |
| Visceral pleural invasion | Positive | 5.32  (2.41–11.7) | <0.001 |  | 3.43  (1.57–7.52) | 0.002 |
| STAS | Positive | 1.43  (0.65–3.13) | 0.37 |  |  |  |

CI, Confidence interval; HR, Hazard ratio; NA, Not applicable; STAS, Spread through air space.

**Supplementary Table S8.** Rate of patients with recurrence according to each margin distance standards

| Predominant pattern | Margin distance standard | Recurrence rate | |
| --- | --- | --- | --- |
|  |  | Locoregional recurrence | Local recurrence |
| GGO-predominant  (N = 40) | < 20 mm (N = 31) | 0% | 0% |
|  | ≥ 20 mm (N = 9) | 0% | 0% |
|  | < Whole tumor size (N = 37) | 0% | 0% |
|  | ≥ Whole tumor size (N = 3) | 0% | 0% |
| Solid-predominant  (N = 125) | < 20 mm (N = 98) | 22%, 22/98 | 10%, 10/98 |
|  | ≥ 20 mm (N = 27) | 19%, 5/27 | 11%, 3/27 |
|  | < Whole tumor size (N = 113) | 21%, 24/113 | 11%, 12/113 |
|  | ≥ Whole tumor size (N = 12) | 25%, 3/12 | 8%, 1/12 |

Recurrence rate is shown as percentage, the number of patients with recurrence/ the number of patients obtaining each margin distance standard.

GGO, ground glass opacity.

**Supplemental Table S9.** Validating analysis using lymph node dissected cases (N = 111)

| Variables | | Univariate analysis | | Multivariate analysis | | |
| --- | --- | --- | --- | --- | --- | --- |
|  |  | HR  (95% CI) | p-value |  | Adjusted HR (95% CI) | p-value |
| **Disease-free survival** | |  | |  |  | |
| Predominant pattern | Solid-predominant | 4.10  (1.60–10.47) | 0.003 |  | 3.15  (1.19–8.32) | 0.02 |
| Tumor histology | Adenocarcinoma | 3.16  (1.68–5.92) | <0.001 |  | 2.87  (1.52–5.42) | 0.001 |
| Visceral pleural invasion | Positive | 3.10  (1.65–5.79) | <0.001 |  | 2.82  (1.50–5.32) | 0.001 |
| **Locoregional recurrence** | |  | | | | |
| Predominant pattern | Solid-predominant | ∞ | 0.003 |  | ∞ | <0.001 |
| Lymph node metastasis | Positive | N.A. | <0.001 |  |  |  |
| Visceral pleural invasion | Positive | 4.13  (1.40–12.2) | 0.01 |  | 3.0  (1.02–8.81) | 0.05 |
| **Local recurrence** | |  | | | | |
| Predominant pattern | Solid-predominant | ∞ | <0.001 |  | ∞ | <0.001 |
| Lymph node metastasis | Positive | N.A. | <0.001 |  |  |  |
| Visceral pleural invasion | Positive | 2.22  (0.38–12.9) | 0.38 |  |  |  |

**^a^** Cox proportional hazards model

**^b^** Fine–Gray competing risk analysis

CI, Confidence interval; HR, Hazard ratio; NA, Not applicable
